# Global research trends and emerging themes in osteosarcoma metabolomics: a bibliometric and visualization analysis

**DOI:** 10.3389/fmolb.2025.1608743

**Published:** 2025-10-24

**Authors:** Haoran Wu, Kunyun Xu, Centao Liu, Heng’an Ge, Jianfeng Yan

**Affiliations:** ^1^ Department of Sports Medicine, Tongji Hospital, School of Medicine, Tongji University, Shanghai, China; ^2^ Department of Orthopedics, Guangxin District People’s Hospital, Shangrao, Jiangxi, China; ^3^ Department of Orthopedics, Affiliated Changshu Hospital to Nantong University, Changshu No. 2 Hospital, Changshu, Jiangsu, China

**Keywords:** metabolomics, osteosarcoma, bibliometrics analysis, chemoresistance, biomarkers

## Abstract

**Introduction:**

This study conducts a systematic bibliometric analysis of the global research landscape of metabolomics in osteosarcoma, aiming to identify research trends, knowledge structures, and emerging directions in the field.

**Methods:**

Publications related to osteosarcoma and metabolomics were retrieved from the Web of Science Core Collection. Bibliometric analysis was performed using CiteSpace, VOSviewer, and Bibliometrix to examine publication trends, geographic and institutional collaborations, author networks, keyword co-occurrence, clustering, and co-citation patterns.

**Results:**

A total of 1,188 eligible articles published between 1995 and 2024 were included. The analysis revealed significant growth in publications and citations over the past decade, with China being the leading contributor. High-frequency keywords such as “biomarkers,” “prognosis,” and “chemoresistance” indicated a strong research focus on tumor progression and treatment resistance. Clustering and burst detection highlighted emerging topics, including extracellular vesicles, microRNAs, and immune metabolism. Co-citation analysis established a knowledge foundation centered on molecular profiling and translational research, with growing interest in spatial and single-cell metabolomics reflecting a shift toward high-resolution metabolic characterization.

**Discussion:**

This bibliometric study underscores the evolving research priorities and methodological advancements within osteosarcoma metabolomics. It offers a comprehensive reference for researchers to understand thematic evolution, recognize knowledge gaps, and foster the development of more precise and integrated metabolic strategies for improving diagnosis and treatment.

## Introduction

Osteosarcoma is the most common primary malignant bone tumor in adolescents, marked by aggressive growth, early metastasis, and limited treatment efficacy in advanced cases (Harris and Hawkins, 2022; Yu and Yao, 2024). Although multimodal therapy combining surgery and chemotherapy has improved outcomes for localized disease, the 5-year survival rate for patients with metastatic or recurrent osteosarcoma remains below 30% (Gianferante et al., 2017). Increasing evidence highlights metabolic reprogramming as a key driver of tumor progression, therapy resistance, and immune evasion in osteosarcoma (Ren et al., 2025; Tian et al., 2023; Zhang et al., 2023; Zhang et al., 2025). Tumor cells exhibit enhanced aerobic glycolysis and glutamine metabolism to support rapid proliferation and survival under stress (Zeng et al., 2023). Excessive lactate production alters the tumor microenvironment, promoting matrix degradation and immune suppression, which facilitates metastasis (Gao et al., 2016; Ren et al., 2020). Functional studies confirm that targeting metabolic enzymes such as LDHA and GLS suppresses tumor growth and metastasis, indicating their therapeutic relevance (Jiang et al., 2021; Mohás et al., 2022). Furthermore, chemoresistance has been linked to metabolic plasticity, including a shift toward fatty acid oxidation, as well as microenvironmental interactions, such as lactate exchange with cancer-associated fibroblasts (CAFs) (Almeida et al., 2023; Griffin et al., 2023; Yang et al., 2023). Recent single-cell metabolomics data also implicate aspartate metabolism in drug-resistant subpopulations (Cheng et al., 2024).

While metabolic targeting has led to promising therapies in other cancers - such as IDH inhibitors in mutant acute myeloid leukemia - similar progress in osteosarcoma remains limited (Amaya and Pollyea, 2018; Liu and Gong, 2019). The disease’s metabolic complexity and lack of validated biomarkers hinder clinical translation (Gill et al., 2013). Nonetheless, these findings underscore the potential of metabolism-focused approaches in osteosarcoma. In this context, metabolomics provides a powerful platform to uncover metabolic vulnerabilities, guide therapeutic strategies, and identify biomarkers.

Metabolomics, as a high-throughput strategy to profile low-molecular-weight metabolites in biological systems, offers a direct readout of cellular biochemical activity and has become an essential tool in cancer research (Rinschen et al., 2019; Wishart, 2016). Metabolomics has been instrumental in characterizing tumor-specific metabolic phenotypes, identifying actionable vulnerabilities, and discovering predictive biomarkers (Kaushik and DeBerardinis, 2018). In breast and prostate cancers, serum metabolite panels have been developed to aid in early diagnosis and risk stratification (Asiago et al., 2010). For instance, phospholipid and amino acid signatures have been shown to distinguish molecular subtypes and predict treatment response (Lee and Lam, 2025; Lucarelli et al., 2015). In gliomas, the identification of 2-hydroxyglutarate as an oncometabolite produced by mutant IDH1 not only provided a diagnostic marker but also led to the development of FDA-approved IDH-targeted therapies (Baek et al., 2024; Xu et al., 2019). These cases exemplify how metabolomics can bridge basic metabolic insights with translational applications.

Moreover, advances in spatial and single-cell metabolomics have further refined our understanding of metabolic heterogeneity within tumors, enabling the dissection of treatment-resistant subpopulations and microenvironmental interactions (Chen et al., 2024; Rappez et al., 2021). These technologies are particularly relevant to osteosarcoma, a cancer known for its intratumoral heterogeneity and variable clinical behavior. Despite its clinical importance, osteosarcoma remains underexplored in the context of metabolomics compared to more common solid tumors. Leveraging metabolomic approaches in osteosarcoma may therefore uncover new biomarkers, therapeutic targets, and mechanistic insights that have been overlooked by conventional genomic or proteomic studies.

Despite growing interest in tumor metabolism, the application of metabolomics in osteosarcoma remains relatively limited. Recent studies have begun to uncover distinctive metabolic features of osteosarcoma cells. Enhanced glycolytic activity and elevated lactate production have been linked to increased metastatic potential, with lactate acting not only as a metabolic byproduct but also as a signaling molecule that reshapes the tumor microenvironment and promotes immune evasion (Chen et al., 2023; Payen et al., 2020; Pereira-Nunes et al., 2020). Additionally, osteosarcoma cells demonstrate a strong dependence on glutamine metabolism. Upregulation of glutaminase (GLS) has been associated with increased resistance to chemotherapeutic agents such as cisplatin and methotrexate, and GLS inhibition has shown promise in sensitizing tumors to treatment (Chen P. et al., 2021; Wu et al., 2023). Recent single-cell metabolomics studies have identified enriched aspartate metabolism in drug-resistant osteosarcoma subpopulations, offering new mechanistic insights into intratumoral heterogeneity and resistance (Cheng et al., 2024).

Despite these advances, several limitations hinder the development of metabolomics-informed strategies in osteosarcoma. Most existing studies are exploratory in nature, based on small, single-center cohorts, and lack validation across diverse clinical populations. Moreover, findings are often fragmented, focusing on isolated metabolic pathways without integrating systemic metabolic networks or their interaction with genomic and immunologic profiles. There is also a lack of standardized analytical pipelines and shared metabolomics datasets, which limits reproducibility and cross-study comparison. These gaps collectively impede the translation of metabolic findings into robust clinical biomarkers or therapeutic targets.

Given the complexity and heterogeneity of osteosarcoma, a comprehensive, data-driven understanding of current research efforts is urgently needed. Mapping the global research landscape can help identify core research clusters, uncover underexplored areas, and guide future directions with greater precision. In this context, bibliometric analysis provides a valuable framework to systematically evaluate research output, track thematic evolution, and highlight emerging trends in the field (Bai et al., 2025).

This study aims to perform a comprehensive bibliometric analysis of global literature on metabolomics in osteosarcoma. We seek to summarize publication trends, identify major contributors and collaborative networks, detect research hotspots and evolving methodologies, and highlight underexplored areas. This work is intended to provide a data-driven foundation for advancing precision research, fostering interdisciplinary collaboration, and accelerating clinical translation in the field of osteosarcoma metabolism.

## Materials and methods

### Data source and retrieval strategy

To ensure the reliability and academic rigor of the bibliometric analysis, all publication data in this study were obtained from the Web of Science Core Collection (WoSCC), a widely acknowledged source for high-quality bibliographic information in scientific research. This database was selected for its comprehensive indexing of peer-reviewed journals and its compatibility with mainstream bibliometric tools. A systematic search was conducted on 12 December 2024, encompassing publications from the inception of the database until the date of retrieval. The search strategy was carefully constructed to encompass a broad range of metabolomics-related studies within the context of osteosarcoma. The search formula was defined as follows: (TS = (“metabolomics” OR “metabolites” OR “metabolic profiling” OR “biomarkers” OR “metabolic pathways” OR “metabolic markers”)) AND TS = (“osteosarcoma” OR “bone sarcoma” OR “bone cancer” OR “osseous sarcoma” OR “central osteosarcoma” OR “medullary osteosarcoma” OR “sclerosing osteosarcoma” OR “subtype osteoblastic osteosarcoma” OR “chondroblastic osteosarcoma” OR “fibroblastic osteosarcoma”).

The initial search yielded 1,260 publications. To ensure relevance and consistency, only articles and review papers written in English were retained for further analysis. After eliminating duplicates using the built-in deduplication function of CiteSpace (version 6.2. R4), a total of 1,188 unique records were finalized for subsequent processing.

### Inclusion criteria and data preparation

Publications were included based on the following criteria: (1) the study explicitly investigated the application of metabolomics or related metabolic analyses in the context of osteosarcoma; (2) the document type was limited to original research articles or review papers; and (3) the publication language was English. Exclusion criteria included conference abstracts, editorial materials, corrections, and non-peer-reviewed content.

The downloaded records were saved in plain text format and imported into CiteSpace for format conversion and data standardization. Metadata fields such as titles, abstracts, keywords, author information, institutional affiliations, and cited references were extracted to support multilevel bibliometric analysis.

### Data analysis and visualization tools

To explore the intellectual structure and thematic evolution of metabolomics research in osteosarcoma, we employed a combination of bibliometric software tools, including CiteSpace (v6.2. R4), VOSviewer (v1.6.18), and the Bibliometrix R package.

CiteSpace was utilized to generate time-sliced knowledge maps, detect reference citation bursts, and visualize the temporal evolution of research themes. The parameters applied were: time slicing from 2000 to 2024 with 1-year intervals; term sources set to titles, abstracts, author keywords, and keywords plus; selection criteria of Top N = 50 per time slice; and clustering resolution parameter k = 15. The citation network analysis identified influential references and shifting research emphases over time.

VOSviewer was applied to construct visual maps of co-authorship, institutional collaboration, keyword co-occurrence, and journal co-citation networks. Node size was proportional to publication or occurrence frequency, while inter-node links represented the strength of associations. Thematic clusters were color-coded to differentiate major research areas.

Bibliometrix, an R-based open-source framework, was employed for complementary statistical analyses and trend visualizations. This included annual publication output, most productive countries and institutions, highly cited papers, and keyword growth dynamics.

### Ethical statement

This study did not involve human or animal subjects and was entirely based on secondary data retrieved from a publicly accessible database. Therefore, no institutional ethical approval was required.

## Results

### Publication and citation trends over time

**FIGURE 1:**
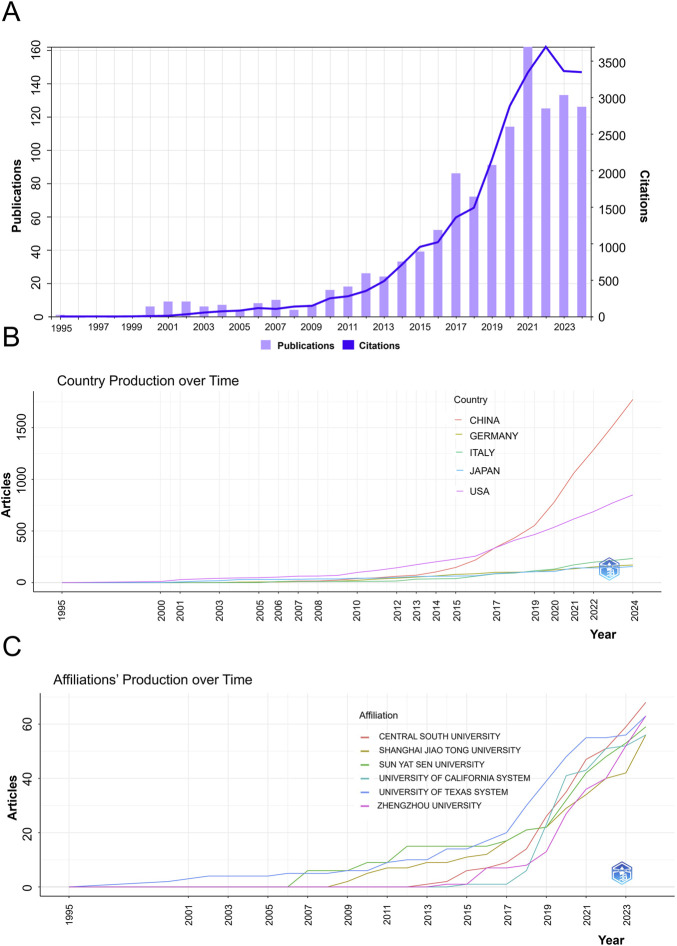
Trend chart of annual publication volume and citation frequency. **(A)** Annual trend in publication volume and citation frequency; **(B)** Timeline of countries; **(C)** Trend of publications by institution over time.

To evaluate the temporal evolution of research activity in the field of metabolomics in osteosarcoma, we analyzed the annual number of publications and total citations from 1995 to 2024 (Figure 1). The analysis reveals a clear and sustained upward trend in both indicators, highlighting the growing global academic interest in this interdisciplinary domain.

During the initial stage (1995–2009), research activity remained modest, with fewer than 10 publications per year and minimal citation impact. This period reflected the exploratory phase of integrating metabolomics into osteosarcoma research. From 2010 onward, the field entered a phase of accelerated growth, with the annual publication count increasing significantly-from 10 in 2010 to 134 in 2024. The most prolific year was 2021, with 161 published articles.

Citation frequency demonstrated a parallel upward trajectory, particularly from 2015 onwards. The number of citations peaked in 2022, surpassing 3,600 annual citations. Although citation counts slightly declined in the following years (2023–2024), they remained at a high level (>3,000 citations annually), indicating the continued relevance and influence of previously published work.

Taken together, these data suggest that metabolomics has emerged as a key research avenue in osteosarcoma over the past decade. The sharp increase in publication volume and citation frequency reflects both the expansion of methodological applications and the growing recognition of metabolic reprogramming as a hallmark of osteosarcoma pathophysiology.

### Global distribution and collaboration patterns among countries

**FIGURE 2:**
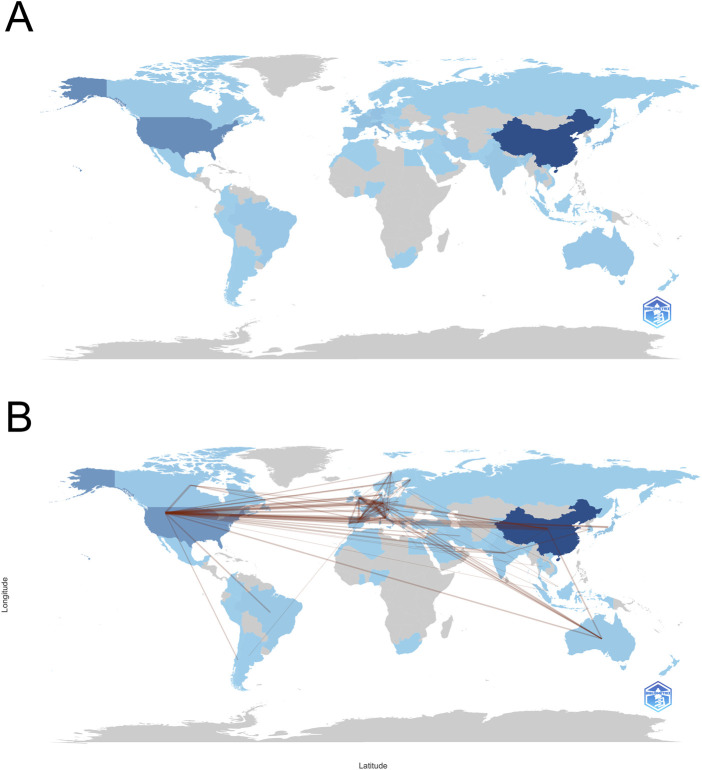
**(A)** Country scientific production; **(B)** Country collaboration map.

**FIGURE 3:**
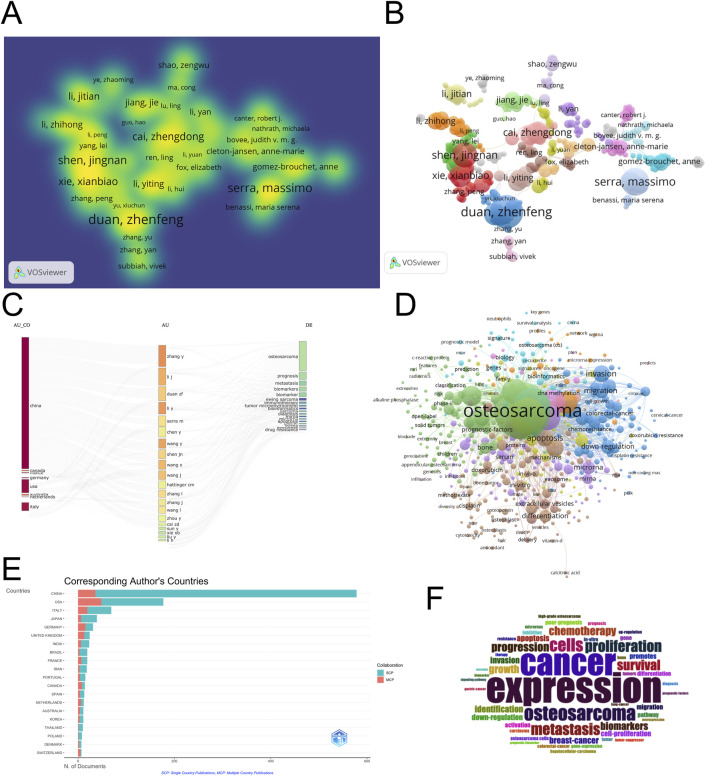
Country production over time. **(A)** Network visualization of author collaborations; **(B)** Network visualization of author collaborations; **(C)** Statistical chart of authors; **(D)** Network visualization of keyword co-occurrence; **(E)** Statistical chart of authors countries; **(F)** Keyword tag cloud.

To explore the global research landscape of metabolomics in osteosarcoma, a country-level collaboration network and longitudinal productivity analysis were conducted (Figures 2, 3). A total of 68 countries have contributed to this field, indicating wide international engagement.

As shown in Figure 3 and detailed in Table 1, China emerged as the most prolific country, accounting for 599 publications since its initial contribution in 2006. This is followed by the United States (n = 241, since 2000) and Italy (n = 84, since 2004). Notably, China has demonstrated a dramatic increase in output, surpassing all other countries by a wide margin after 2018. The United States and Japan showed steady productivity throughout the 2 decades, whereas European countries such as Germany, the United Kingdom, and Italy exhibited moderate but consistent contributions. Countries like India and France entered the field later but have shown growing momentum.

**TABLE 1:** Statistics of publication frequency by country.

No.	Publication volume	Centrality	Year of publication	Country
1	599	0	2006	PEOPLES R CHINA
2	241	0.06	2000	United States
3	84	0.12	2004	ITALY
4	55	0.24	2002	GERMANY
5	47	0	2000	JAPAN
6	40	0.5	2000	ENGLAND
7	33	0.2	2010	INDIA
8	30	0.1	2007	FRANCE
9	27	0.59	2004	NETHERLANDS
10	25	0.17	2005	CANADA

In terms of network centrality - a proxy for the importance of a country in facilitating international research collaboration - several countries demonstrated high values despite relatively fewer publications. For instance, the Netherlands (centrality = 0.59), the United Kingdom (0.50), and Germany (0.24) play key bridging roles in the global research network (Table 1). In contrast, China, despite its dominant publication volume, shows a centrality score of 0.00, suggesting that its international collaboration density remains limited.

The geographical collaboration map (Figure 2) further highlights that the United States acts as a major hub of international cooperation, particularly with European and Asian countries. While China’s connections have expanded, its collaboration appears more regional and less diversified. These findings imply that strengthening cross-national cooperation, especially involving highly central countries, may further enhance knowledge integration and accelerate global advancement in osteosarcoma metabolomics research.

### Institutional contributions and productivity trends

**FIGURE 4:**
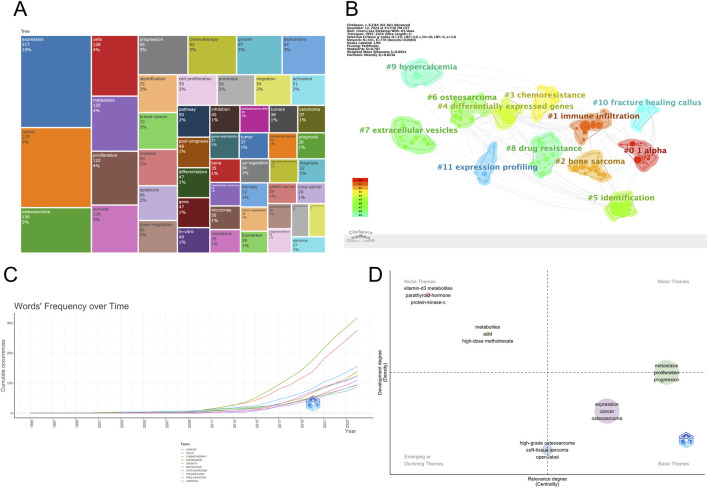
Changes in publications by institutions over time. **(A)** Keyword tree map; **(B)** Keyword clustering diagram; **(C)** Keyword frequency change over time; **(D)** Keyword clustering diagram.

To further elucidate the organizational landscape of research on metabolomics in osteosarcoma, we examined the publication output and centrality of the top ten most productive institutions (Figure 4; Table 2). Chinese research institutions dominated the top ranks, with Central South University leading at 35 publications since its entry in 2013, followed closely by Sun Yat-sen University (29 publications, since 2007) and Shanghai Jiao Tong University (28 publications, since 2017). Other notable Chinese institutions include Zhengzhou University, Fudan University, Jilin University, and Nanchang University, reflecting China’s widespread academic investment in this area. Internationally, the University of Texas System (27 publications) and the University of California System (18 publications) emerged as key contributors from the United States, along with the National Institutes of Health (NIH). Among these, the University of Texas System exhibited the highest centrality score (0.18), indicating its stronger role in collaborative networks.

**TABLE 2:** Statistics of publication frequency of the top ten research institutions.

No.	Publication volume	Centrality	Years of publication	Agency
1	35	0.01	2013	Central South University
2	29	0.15	2007	Sun Yat Sen University
3	28	0	2017	Shanghai Jiao Tong University
4	27	0.18	2000	University of Texas System
5	24	0	2016	Zhengzhou University
6	23	0.04	2006	Fudan University
7	18	0.03	2018	University of California System
8	18	0.01	2004	National Institutes of Health (NIH) - United States
9	17	0.01	2017	Jilin University
10	15	0.01	2021	Nanchang University

The institutional production timeline (Figure 4) reveals that most of the leading Chinese institutions began contributing significantly only after 2015, yet quickly accelerated their output. In contrast, U.S. institutions like the University of Texas System showed earlier and more sustained engagement since the early 2000s. Central South University exhibited the steepest upward trend in the most recent years, surpassing its peers by 2024.

Despite high publication output, many institutions displayed low betweenness centrality (<0.05), suggesting relatively weak roles in inter-institutional collaboration. This observation highlights a current structural limitation: while regional productivity is strong - especially in China - global integration across institutions remains limited. These results emphasize the need to foster more international and cross-institutional cooperation to bridge fragmented efforts and enhance knowledge exchange in this rapidly evolving field.

### Author collaboration networks and core contributors

**FIGURE 5:**
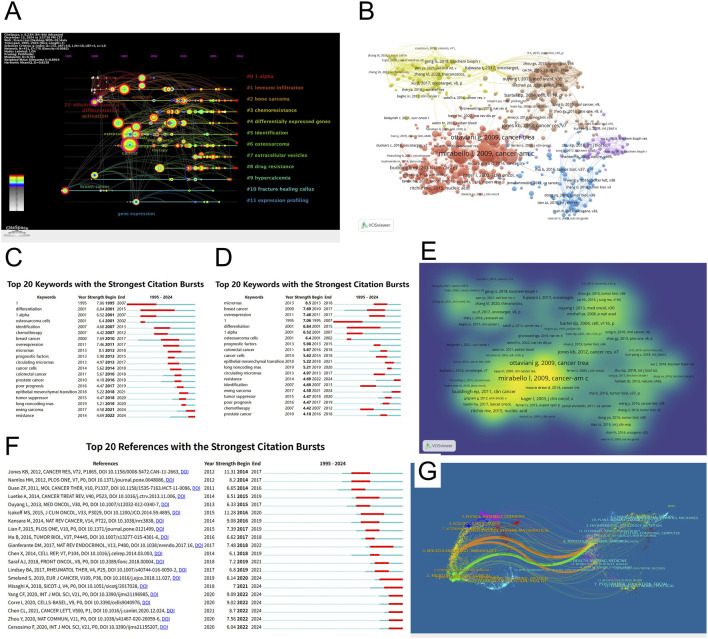
Author collaboration network. **(A)** Timeline visualization of keyword clusters; **(B)** Citation co-occurrence network; **(C)** Keyword burst chart arranged by time; **(D)** Arrange the key words in the keyword emergence chart according to their emergence degree; **(E)** Document density visualization; **(F)** Cited reference burst diagram; **(G)** Biplot overlay of scientific journals.

To identify influential researchers and examine collaborative structures in the field, an author co-authorship network and density visualization were constructed using VOSviewer. A total of 791 authors who had published at least two articles were included in the analysis. As shown in Figure 5A, the co-authorship network reveals several well-defined collaborative clusters, with the node size representing the number of publications and the distance between nodes indicating the strength of co-authorship. Notably, Duan Zhenfeng, Serra Massimo, Cai Zhengdong, and Shen Jingnan emerged as central figures in the field, each anchoring a distinct subnetwork of collaboration. The corresponding density map (Figure 5B) further highlights their prominence, with brighter regions reflecting higher publication density and collaborative intensity. According to Table 3, Duan Zhenfeng led the author ranking with 12 publications and a total citation count of 436. Serra Massimo followed with 10 publications but had the highest citation frequency among the top authors (521), suggesting greater influence per article. Shen Jingnan and Cai Zhengdong also showed strong performance, with 9 and 8 publications respectively and high connection strength values (61 and 48), indicating their pivotal roles in shaping collaborative research efforts. The collaborative landscape is characterized by moderate fragmentation, with regional clusters - particularly those led by Chinese and European researchers - tending to collaborate more intensively within their respective networks. This pattern underscores the importance of enhancing interregional collaboration to further diversify perspectives and methodologies in the field. Overall, these findings suggest that a small number of key researchers have driven much of the scholarly output and influence in osteosarcoma metabolomics. Promoting broader and more integrated author collaborations may help to consolidate expertise and accelerate research innovation across institutional and geographic boundaries.

**TABLE 3:** Frequency of publications by the top ten authors.

No.	Publication frequency	Citation frequency	Connection strength	Author’s name
1	12	436	47	duan, zhenfeng
2	10	521	42	serra, massimo
3	9	226	61	shen, jingnan
4	8	336	48	cai, zhengdong
5	8	125	31	min, li
6	8	215	59	xie, xianbiao
7	7	300	47	hua, yingqi
8	7	59	23	li, jitian
9	7	141	36	man, tsz-kwong
10	7	187	36	picci, piero

**FIGURE 6:**
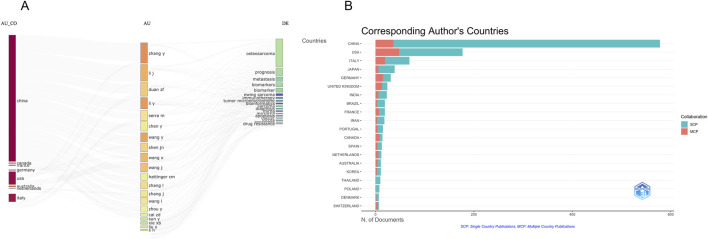
Statistical chart of authors and their countries. **(A)** Author analysis diagram; **(B)** Country distribution of authors.

To better understand the geographic origins of influential research and the thematic roles of corresponding authors, we conducted an in-depth analysis of author-country affiliations and their associated research focuses. Figure 6A presents a three-field plot linking authors’ countries (AU_CO), individual authors (AU), and high-frequency keywords (DE), while Figure 6B visualizes the distribution of corresponding authors across countries and distinguishes between single-country publications (SCP) and multi-country publications (MCP). As shown in Figure 6B, China dominates the field in terms of corresponding author output, contributing over 600 publications, far surpassing the United States and Italy. However, most of China’s publications are categorized as SCPs, indicating limited international collaboration. In contrast, countries such as the United States, Germany, and the United Kingdom demonstrated a relatively higher proportion of MCPs, reflecting stronger involvement in global partnerships. These findings confirm that while Chinese scholars contribute substantially to the literature in terms of quantity, international scholars - especially those from Europe and North America - tend to be more integrated into collaborative and translational research efforts. Furthermore, the convergence of Chinese and international authors on common keywords underscores the global relevance of key challenges such as drug resistance and biomarker identification.

### Keyword Co-occurrence and research focus evolution

**FIGURE 7:**
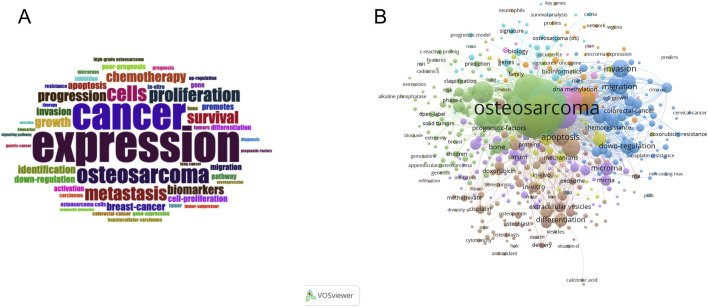
**(A)** Keyword word cloud; **(B)** Keyword co-occurrence network map.

To identify research hotspots and thematic directions in the field of metabolomics in osteosarcoma, a comprehensive keyword analysis was conducted based on co-occurrence frequency, network density, and temporal trends. The word cloud visualization in Figure 7A provides an intuitive overview of high-frequency terms, with larger font sizes indicating more frequent usage. Keywords such as “expression” “cancer” “osteosarcoma” “metastasis” “biomarkers” “cells” and “proliferation” were the most prominent. These terms align closely with core topics in tumor biology, suggesting a predominant focus on molecular mechanisms, disease progression, and biomarker identification. The keyword co-occurrence network in Figure 7B further illustrates the structural connectivity among terms. “Osteosarcoma” was the central node (co-occurrence frequency: 715; total link strength: 5,267), connecting with a dense web of terms related to pathogenesis, treatment strategies, and molecular diagnostics. Notably, clusters emerged around “apoptosis” “drug resistance” “extracellular vesicles” and “epithelial - mesenchymal transition” indicating specialized subfields with increasing scholarly attention. Quantitative data in Table 4 confirm the centrality and frequency of key terms. “Cancer” (n = 324, link strength = 2,416), “expression” (n = 319, link strength = 2,477), “biomarkers” (n = 180), and “metastasis” (n = 179) ranked among the top thematic keywords, reflecting continued emphasis on diagnostic and prognostic indicators in osteosarcoma.

**TABLE 4:** Co-occurrence frequency statistics of the top ten keywords.

No.	Keywords	Frequency	Connection strength
1	osteosarcoma	715	5,267
2	cancer	324	2,416
3	expression	319	2,477
4	biomarkers	180	1,464
5	metastasis	179	1,521
6	prognosis	175	1,439
7	proliferation	139	1,182
8	Cells	138	990
9	Biomarkers	124	1,031
10	survival	120	943
11	chemotherapy	107	819
12	progression	99	868
13	apoptosis	90	687
14	Growth	87	674
15	Invasion	84	718

**FIGURE 8:**
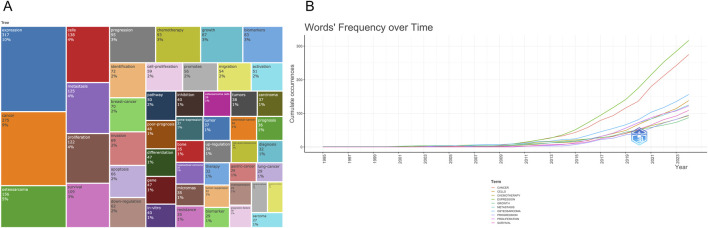
**(A)** Keyword tree map; **(B)** Keyword frequency change over time.

The treemap diagram (Figure 8A) visualizes keyword categories based on frequency and hierarchical structure. Categories such as “cancer” “expression” “cells” and “proliferation” occupied the largest blocks, reinforcing their central importance. Meanwhile, topics like “drug resistance” “microRNAs” and “tumor suppressor genes” emerged as smaller but growing areas. Temporal trends in Figure 8B show a sharp rise in cumulative usage of terms such as “cancer” “osteosarcoma” “expression” and “biomarkers” since 2015. Terms like “microRNAs” “exosomes” and “chemotherapy” also showed accelerated growth in recent years, suggesting an expanding interest in non-coding RNA regulation and treatment-related metabolic dynamics. Collectively, these keyword analyses reveal that the field is evolving from basic molecular characterization toward more integrated themes such as therapeutic response, tumor microenvironment, and personalized biomarker development. The diversity and convergence of keyword clusters also indicate an increasing complexity and maturity in the research landscape.

### Keyword clustering and thematic evolution

**FIGURE 9:**
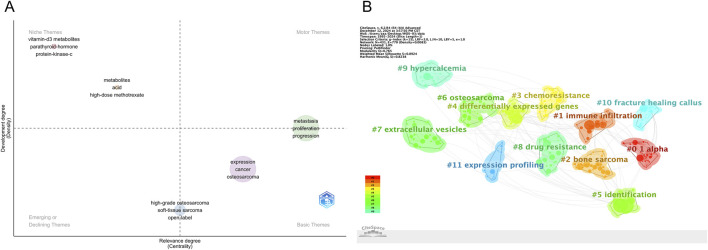
**(A,B)** Keyword clustering map.

To further clarify the structural composition and developmental trajectory of research topics in the field, keyword co-occurrence clustering analysis was performed using CiteSpace. A total of 12 clusters were identified based on the log-likelihood ratio (LLR) algorithm, and their quality was assessed using the modularity index (Q = 0.765) and silhouette coefficient (S = 0.8924), indicating high clustering reliability and structural validity (Figure 9; Table 5). Each cluster was assigned a representative label based on its most frequent or distinguishing terms. The largest clusters were #0 “1 alpha” (n = 41, S = 0.937), #1 “immune infiltration” (n = 40, S = 0.889), and #2 “bone sarcoma” (n = 34, S = 0.907). Other prominent clusters included #3 “chemoresistance”, #4 “differentially expressed genes”, #7 “extracellular vesicles”, and #8 “drug resistance”, all of which demonstrated strong internal consistency (S > 0.86).

**TABLE 5:** Cluster information statistics.

No.	Cluster labels	Size	Silhouette coefficient
1	#0 1 alpha	41	0.937
2	#1 Immune Infiltration	40	0.889
3	#2 bone sarcoma	34	0.907
4	#3 chemoresistance	33	0.899
5	#4 differentially	33	0.86
6	#5 identification	33	0.921
7	#6 osteosarcoma	32	0.772
8	#7 Extracellular vesicles	29	0.911
9	#8 drug resistance	27	0.905
10	#9 hypercalcemia	26	0.89
11	#10 fracture healing callus	22	0.984
12	#11 expression profiling	16	0.933

**FIGURE 10:**
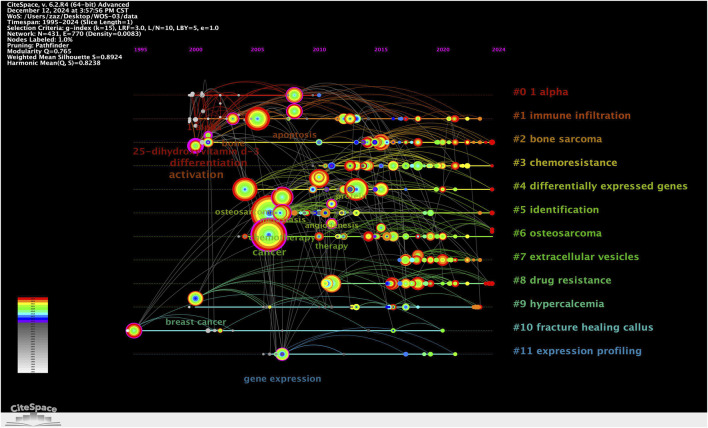
Keyword clustering timeline map.

The strategic thematic map (Figure 9A) provides a quadrant-based classification of research themes according to their centrality (relevance) and density (development). Clusters such as “metastasis” “proliferation” and “progression” were identified as motor themes, characterized by both high centrality and high density, indicating well-developed and influential areas. In contrast, topics such as “high-grade osteosarcoma” “epithelioid” and “soft tissue sarcoma” appeared in the emerging or declining quadrant, suggesting either nascent or waning interest. Themes like “expression” and “cancer” were positioned as basic themes, fundamental to the field but with room for further thematic enrichment. The timeline visualization in Figure 10 illustrates the temporal evolution of each cluster, showing both onset time and sustained activity. Clusters such as #11 “expression profiling” and #0 “1 alpha” appeared as early as the mid-1990s, indicating foundational roles in the field’s early development. More recent clusters, such as #7 “extracellular vesicles”, #3 “chemoresistance”, and #8 “drug resistance”, demonstrated heightened activity in the past decade, suggesting they represent frontier research areas. Notably, cluster #10 “fracture healing callus” - despite its relatively small size (n = 22) - showed the highest silhouette coefficient (S = 0.984), indicating an exceptionally coherent thematic identity. This may reflect a specialized subfield focused on metabolic aspects of bone repair and regeneration in the osteosarcoma context.

Overall, the clustering results demonstrate a multidimensional and temporally dynamic research landscape. While foundational themes such as gene expression and molecular profiling remain central, there is a clear shift toward applied and translational topics, including immune regulation, extracellular communication, and therapeutic resistance. These trends underscore the field’s maturation and expanding complexity, as metabolomics increasingly intersects with immunology, pharmacogenomics, and personalized medicine.

### Burst detection analysis of keywords

**FIGURE 11:**
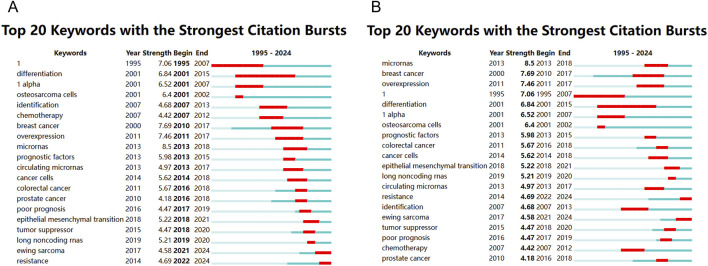
Keyword emergence graph (sorted by time and by emergence). **(A)** Top 20 keywords with the strongest citation bursts; **(B)** Top 20 keywords with the strongest citation bursts.

To trace the temporal dynamics of research priorities and identify emerging topics, a burst detection analysis of keywords was performed using CiteSpace. Citation bursts highlight terms that experience a sharp increase in frequency over a defined period, reflecting heightened attention in the academic community. As shown in Figure 11A, from 2007 onwards, a noticeable shift occurred toward applied clinical and diagnostic themes. Keywords like “identification” (4.68), “chemotherapy” (4.18), “microRNAs” (8.5), and “prognostic factors” (5.98) began to dominate the landscape. The emergence of “microRNAs” and “circulating microRNAs” (4.97) between 2013–2018 and 2013–2017, respectively, reflects the growing interest in non-coding RNA as metabolic biomarkers and regulators of therapeutic response. More recent bursts (2018–2024) point to the field’s increasing sophistication and diversification. Noteworthy terms include “epithelial mesenchymal transition” (5.22), “ewing sarcoma” (4.58), and “resistance” (4.69), which reflect ongoing concerns with tumor aggressiveness, histological subtypes, and chemoresistance mechanisms. These keywords are not only timely but also clinically relevant, marking a transition from descriptive profiling toward mechanistic and translational investigations. Figure 11B reorganizes the top 20 keywords by burst intensity and reaffirms “microRNAs” (Strength = 8.5) as the most prominent emergent topic, followed by “breast cancer” (7.69) and “overexpression” (7.46). The clustering of bursts around 2013–2020 suggests that this period was particularly active in introducing molecular biology concepts into osteosarcoma metabolomics research.

### Co-citation analysis and intellectual foundation

**FIGURE 12:**
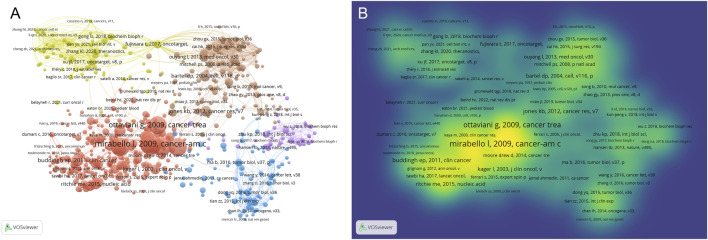
Literature co-citation analysis and density visualization. **(A)** Analysis of co-citations in literature; **(B)** Density visualization diagram.

To uncover the intellectual structure and foundational knowledge base of metabolomics research in osteosarcoma, co-citation analysis was conducted using VOSviewer and CiteSpace. This approach identifies frequently cited references that underpin current research and highlights papers that have triggered significant academic attention over time. As shown in Figure 12A, the co-citation network displays several distinct clusters, each representing a thematic knowledge domain. The largest and most densely connected cluster centers around landmark studies by Ottaviani G (2009) and Mirabello L (2009), both of which focus on epidemiological and clinical characteristics of osteosarcoma. These references serve as cornerstones in understanding disease onset, treatment outcomes, and survival statistics. The corresponding density visualization in Figure 12B further highlights these central nodes as the intellectual hubs of the field. Table 6 lists the top 10 most cited references. The most cited paper was authored by Gill J (2021) and published in Nature Reviews Clinical Oncology (38 citations), providing a comprehensive update on therapeutic advancements in osteosarcoma. Smeland S (2019), with 36 citations, offered critical insights from the EURAMOS-1 trial, emphasizing patient stratification and long-term prognosis. Other frequently cited works include contributions by Isakoff MS (2015) on standard treatment protocols and Corre I (2020) on oxidative stress and radiosensitivity, both of which have significantly shaped contemporary therapeutic perspectives.

**TABLE 6:** Statistical frequency table of cited literature.

No.	Citations	Centrality	Cited articles
1	38	0.03	Gill J, 2021, NAT REV CLIN ONCOL, V18, P609, DOI 10.1038/s41571-021–00519-8
2	36	0.01	Smeland S, 2019, EUR J CANCER, V109, P36, DOI 10.1016/j.ejca.2018.11.027
3	33	0.02	Corre I, 2020, CELLS-BASEL, V9, P0, DOI 10.3390/cells9040976
4	33	0.06	Isakoff MS, 2015, J CLIN ONCOL, V33, P3029, DOI 10.1200/JCO.2014.59.4895
5	28	0.05	Gianferante et al. (2017), NAT REV ENDOCRINOL, V13, P480, DOI 10.1038/nrendo.2017.16
6	28	0.04	Harrison DJ, 2018, EXPERT REV ANTICANC, V18, P39, DOI 10.1080/14737140.2018.1413939
7	27	0.06	Heymann MF, 2019, CELL IMMUNOL, V343, P0, DOI 10.1016/j.cellimm.2017.10.011
8	26	0.02	Sayles LC, 2019, CANCER DISCOV, V9, P46, DOI 10.1158/2159-8290.CD-17-1152
9	24	0.01	Yang CF, 2020, INT J MOL SCI, V21, P0, DOI 10.3390/ijms21196985
10	23	0.03	Chen CL, 2021, CANCER LETT, V500, P1, DOI 10.1016/j.canlet.2020.12.024

**FIGURE 13:**
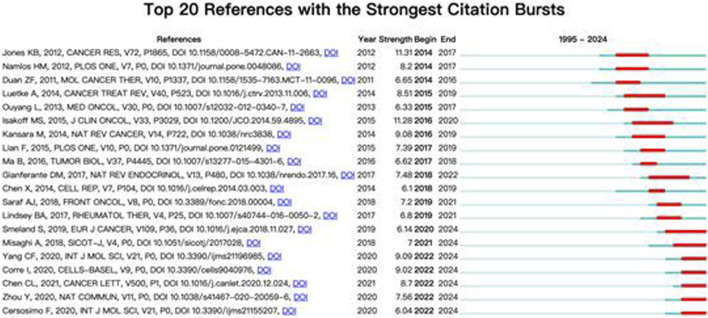
Cited articles burst map.

In terms of betweenness centrality, which reflects a reference’s bridging role in the knowledge network, Gianferante DM (2017) and Heymann MF (2019) exhibited relatively high scores (centrality = 0.06), indicating their integration across multiple subfields such as endocrinology, immunology, and treatment resistance. Complementing these findings, Figure 13 presents the top 20 references with the strongest citation bursts, highlighting literature that has attracted surges of attention during specific periods. For example, Jones KB (2012) experienced the strongest burst (Strength = 11.31, 2014–2017), underscoring its influence during the critical transition to molecular classification and gene expression profiling. More recent bursts include Yang CF (2020) and Feron O (2020), both peaking between 2022 and 2024, reflecting growing interest in tumor metabolism and microenvironmental regulation.

Collectively, the co-citation and burst detection analyses reveal a research foundation rooted in clinical characterization, now progressively shifting toward molecular pathogenesis and therapeutic innovation. These findings not only identify the core literature shaping the field but also map the trajectory of scholarly influence and emerging paradigms.

### Dual-map overlay of journals

**FIGURE 14:**
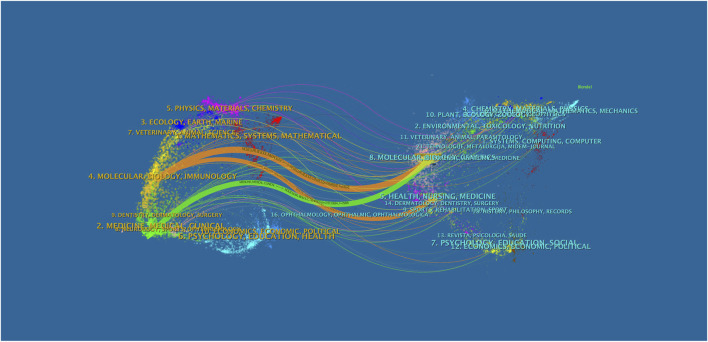
Journal double image overlay.

To visualize the disciplinary diffusion and citation trajectories within the field, a dual-map overlay of journals was generated using CiteSpace. This visualization technique maps the citing and cited journals across different scientific domains and illustrates their interconnections through citation paths. As shown in Figure 14, the left side of the map represents the domains of the citing journals, while the right side reflects those of the cited journals. Colored lines between the two regions indicate the citation flows, highlighting the knowledge transfer from the citing fields to the intellectual bases. The most prominent citation paths originate from journals in Molecular/Biology/Genetics and Medicine/Medical/Clinical, pointing toward cited journals located within Health/Nursing/Medicine, Molecular/Biology/Immunology, and Genetics/Clinical Medicine domains. This pattern underscores the interdisciplinary nature of metabolomics research in osteosarcoma, where experimental molecular findings are frequently cited in translational and clinical medicine contexts. In particular, the robust citation trajectory from Molecular Biology and Genetics to Health and Medicine reflects the central role of omics-based discoveries in informing clinical applications such as biomarker validation, treatment stratification, and prognostic modeling. Additionally, contributions from Environmental/Ecology and Mathematics/Systems domains indicate occasional methodological or systemic approaches being integrated into biomedical frameworks.

Overall, the dual-map overlay illustrates a clear translational flow of knowledge from fundamental biosciences to clinical and healthcare applications, validating the field’s evolution from descriptive metabolite profiling toward actionable clinical insights. The widespread coverage across both life science and medical disciplines also reinforces the highly interdisciplinary character of this research area.

## Discussion

This study provides the first comprehensive bibliometric analysis of global research on the application of metabolomics in osteosarcoma. Over the past 3 decades, the field has witnessed a steady rise in both publication volume and citation frequency, particularly after 2010, reflecting growing scientific interest in metabolic reprogramming in cancer. High-frequency keywords and clustering results indicate research hotspots centered on tumor progression, chemoresistance, and biomarker discovery. The thematic evolution reveals a clear shift from basic molecular profiling to translational topics with clinical relevance. These findings offer a panoramic understanding of the research landscape and highlight key directions for future investigation.

The keyword analysis and thematic clustering provide a multidimensional view of the evolving research focus in osteosarcoma metabolomics. High-frequency terms such as “biomarkers” “prognosis” “apoptosis” “chemotherapy” and “metastasis” (Table 4; Figure 7) collectively underscore the central role of tumor aggressiveness and treatment response in this field. More notably, burst keyword analysis and cluster timelines reveal a pronounced shift toward chemoresistance-related metabolic reprogramming, particularly after 2013 (Figures 10, 11). Clusters such as #3 “chemoresistance”, #8 “drug resistance” and #7 “extracellular vesicles” suggest sustained attention to therapy resistance as a critical barrier to clinical success.

Mechanistically, osteosarcoma chemoresistance is increasingly recognized as a multifactorial process in which metabolomics plays an integrative explanatory role (Kim et al., 2017; Wang et al., 2023). First, tumor cells can exhibit metabolic adaptability, compensating for glycolytic inhibition by upregulating fatty acid oxidation (FAO) (Chen et al., 2022; Lee et al., 2020). Such plasticity allows osteosarcoma cells to maintain ATP production and resist metabolic stress induced by chemotherapy. Recent metabolomic profiling has shown that cisplatin-resistant osteosarcoma cells display enhanced FAO signatures, supporting this adaptive shift (Montopoli et al., 2011). Second, the tumor microenvironment (TME) contributes significantly to resistance (Yang et al., 2024). Cancer-associated fibroblasts (CAFs) have been shown to secrete lactate, which not only acidifies the extracellular milieu but also fosters immune evasion by suppressing cytotoxic T-cell activity and promoting regulatory T-cell infiltration (Wangpaichitr et al., 2021). This process, reflected by emerging keywords like “extracellular vesicles” and “tumor immunity”, links metabolic exchange in the TME with immunomodulatory dynamics that reduce chemotherapy efficacy. Third, epigenetic regulation, particularly m6A RNA methylation, has gained attention as a modulator of metabolic gene expression. Recent studies have revealed that m6A-modified transcripts encode enzymes central to amino acid metabolism and the oxidative stress response (Chen C. et al., 2021; Han et al., 2024; Hu et al., 2025). In osteosarcoma, aberrant m6A patterns are associated with altered glutamine metabolism and resistance to DNA-damaging agents such as cisplatin. Burst keywords such as “microRNAs” “epithelial - mesenchymal transition (EMT)” and “prognostic factors” further reinforce the role of epigenetic and transcriptional control in metabolic remodeling.

The progression from basic metabolic profiling to these multi-level mechanistic investigations illustrates the maturation of the field. Metabolomics has evolved beyond passive observation toward an active tool for uncovering targetable vulnerabilities in chemoresistant tumors. These findings emphasize the necessity for integrated multi-omics approaches that combine metabolomics with transcriptomics, epigenomics, and spatial profiling to decode the complex interplay between metabolism and resistance phenotypes.

While conventional metabolomics has greatly advanced our understanding of metabolic alterations in osteosarcoma, the emergence of new technologies such as spatial metabolomics and single-cell metabolomics offers the potential to resolve previously inaccessible layers of metabolic heterogeneity and tumor microenvironment (TME) interactions (Allam and Coskun, 2024). Our keyword evolution and cluster analysis (Figures 9, 10) showed increasing attention to topics such as “extracellular vesicles,” “immune infiltration,” and “drug resistance,” which inherently involve spatial and cellular diversity—areas where bulk metabolomics is inherently limited.

Spatial metabolomics, typically based on mass spectrometry imaging (MSI), enables *in situ* mapping of metabolite distributions within tissue sections at sub-millimeter resolution (Najumudeen and Vande Voorde, 2025). This approach is particularly relevant to osteosarcoma, a tumor characterized by heterogeneous cell populations, extensive stromal involvement, and areas of necrosis or calcification. Recent studies in soft tissue sarcomas have shown that spatial metabolomic signatures can delineate metabolic niches associated with hypoxia, osteoid formation, or immune exclusion zones. Applying such techniques to osteosarcoma may reveal site-specific vulnerabilities, such as perivascular FAO enrichment or lactate gradients correlating with immunosuppressive regions.

Single-cell metabolomics, although still technically challenging, provides another layer of resolution, allowing interrogation of cell-to-cell variability in metabolic states (Zenobi, 2013). In osteosarcoma, chemoresistant subclones are often embedded within phenotypically similar populations, and their identification requires beyond-average profiling tools. Emerging single-cell methods, including capillary electrophoresis-mass spectrometry (CE-MS) and single-cell isotope tracing, have begun to uncover distinct glutamine utilization patterns and ROS-scavenging capacities in resistant osteosarcoma cells (DeLaney et al., 2018; Steinhauser et al., 2012; Zhang et al., 2020). These methods may aid in functionally identifying minimal residual disease (MRD) niches or predicting relapse risk after neoadjuvant therapy.

Moreover, integrating spatial and single-cell metabolomics with transcriptomic or proteomic layers (i.e., spatial multi-omics) could help contextualize metabolic signatures within immune or angiogenic landscapes. For instance, linking arginine depletion to myeloid-derived suppressor cell (MDSC) expansion, or lactate accumulation to PD-L1 expression, could inform rational combinations of metabolic inhibitors and immunotherapy (Fletcher et al., 2015; Wang et al., 2025). However, the translation of these techniques into osteosarcoma research remains limited due to technical complexity, tissue preparation challenges in calcified tumors, and lack of standardized pipelines. Addressing these gaps requires cross-disciplinary collaboration and the development of osteosarcoma-specific reference atlases that capture the tumor’s spatial and clonal metabolic architecture.

In summary, new-generation metabolomic tools hold great promise for deciphering intra-tumoral heterogeneity and TME dynamics in osteosarcoma. Their application could shift the current research paradigm from population-level observations toward precision metabolic phenotyping, ultimately improving target identification and therapeutic stratification.

To advance the translational impact of metabolomics in osteosarcoma, future research should focus on several key directions. Elucidating resistance mechanisms through metabolic reprogramming - such as shifts toward fatty acid oxidation, lactate-mediated immunosuppression, and epigenetic alterations like m6A methylation - may reveal novel biomarkers and therapeutic vulnerabilities. Integrating multi-omics platforms, including transcriptomics, proteomics, and epigenomics, will help decode the complex interactions between tumor metabolism, microenvironment, and therapeutic response. Emerging technologies such as spatial metabolomics and single-cell metabolic profiling offer new opportunities to dissect intra-tumoral heterogeneity and identify resistant subclones with distinct metabolic phenotypes. In this context, artificial intelligence and machine learning can facilitate pattern recognition and metabolic subtype stratification, enabling precision therapy design. Additionally, targeting the metabolic crosstalk between tumor and stromal cells - via exosomes, cytokine signaling, and immunometabolites - may offer new combination strategies alongside chemotherapy or immunotherapy. Multimodal approaches that incorporate metabolic inhibitors with immune checkpoint blockade or ferroptosis inducers hold promise for enhancing treatment efficacy and overcoming relapse.

This study provides a systematic overview of global research trends, hotspots, and knowledge structures in osteosarcoma metabolomics, yet certain limitations should be acknowledged. The exclusive use of the Web of Science Core Collection may exclude non-English or recently indexed studies, potentially overlooking novel contributions. The retrospective nature of the data may also underrepresent very recent shifts in methodology or clinical translation. Moreover, as a bibliometric study, our analysis lacks integration with experimental or clinical datasets, limiting biological contextualization. Future efforts should aim to incorporate broader data sources and link bibliometric findings with molecular, imaging, and clinical outcome data. This integrative approach will better capture the evolving complexity of osteosarcoma metabolism and accelerate the discovery of clinically actionable metabolic targets.

## Conclusion

This bibliometric analysis offers a comprehensive overview of global research on metabolomics in osteosarcoma, highlighting key contributors, evolving research themes, and methodological advances. The findings reveal a shift from basic metabolic profiling to more refined investigations into chemoresistance, biomarker discovery, and tumor microenvironment interactions. By mapping the current knowledge structure and identifying research gaps, this study provides a valuable reference for future work aiming to develop more integrated and clinically relevant metabolic strategies for osteosarcoma management.

## Data Availability

The original contributions presented in the study are included in the article/supplementary material, further inquiries can be directed to the corresponding authors.
